# The Effect of an Active Break Intervention on Nonspecific Low Back Pain and Musculoskeletal Discomfort during Prolonged Sitting among Young People—Protocol for a Randomized Controlled Trial

**DOI:** 10.3390/jcm13020612

**Published:** 2024-01-22

**Authors:** Magdalena Plandowska, Marta Kinga Labecka, Aleksandra Truszczyńska-Baszak, Maciej Płaszewski, Reza Rajabi, Beata Makaruk, Dorota Różańska

**Affiliations:** 1 Faculty of Physical Education and Health, Jozef Pilsudski University of Physical Education in Warsaw, 21-500 Biala Podlaska, Poland; magdalena.plandowska@awf.edu.pl (M.P.); maciej.plaszewski@awf.edu.pl (M.P.); beata.makaruk@awf.edu.pl (B.M.); dorota.rozanska@awf.edu.pl (D.R.); 2 Faculty of Rehabilitation, Jozef Pilsudski University of Physical Education in Warsaw, 00-968 Warsaw, Poland; aleksandra.truszczynska@awf.edu.pl; 3 Department of Health and Sport Medicine, Faculty of Physical Education and Sport Sciences, University of Tehran, Tehran 1417614411, Iran; reza.rajabi@awf.edu.pl

**Keywords:** exercise, treatment, spine, posture, students, pain

## Abstract

Background: The most recent evidence has shown that the pandemic of COVID-19 caused an increasing problem with spinal pain in the population of teenagers and young adults. This may be explained by prolonged sitting times in flexed positions with electronic devices. Positions maintained for a prolonged time cause overloading of soft tissue and discogenic symptoms. This study aims to evaluate the effectiveness of the active break program in reducing musculoskeletal discomfort and LBP (low back pain) among young people. Methods: This will be a randomized controlled study. The participants will be recruited from Bachelor’s course students of the Physical Education Department aged 18–25 years. The participants will be assigned to an experimental group (with an active break) and a control group. The group with an active break with lumbar and hip extension exercises will be recommended to take a break for every 30 min of sitting. The control group will receive self-care recommendations. The primary outcomes will be pain intensity (Visual Analogue Scale), disability index (Oswestry Disability Index), and perceived musculoskeletal discomfort during prolonged sitting (Borg scale), assessed at baseline and after the intervention, and the Global Perceived Effect, only assessed after the 12-week intervention. The secondary outcome will be a Post-Intervention Questionnaire (a 5-item self-completed questionnaire), only assessed after the 12-week intervention. Results: Our main research outcome—exercise protocols and interventions—will lead to the development of recommendations and protocols for the LBP population. It is important to determine the effect of interventions that are feasible and effective in addressing LBP and perceived musculoskeletal discomfort in young people. Conclusions: This is the first study examining the effect of active breaks with proposed lumbar and hip extension exercises on reducing or decreasing LBP in students based on a search of the literature. Exercises and recommendations will be the basis for developing proprietary preventative and therapeutic programs, which will be implemented in selected educational institutions.

## 1. Background

According to the World Health Organization (WHO), between 60 and 85% of the world’s people—in both developed and developing countries—lead a sedentary lifestyle, making it one of the major but under-resolved public health problems of our time [1]. The most recent evidence has also shown that the situation relating to the COVID-19 pandemic has adversely affected the health of the entire world population [2,3,4,5]. During the COVID-19 pandemic, many countries switched their teaching programs to e-learning, where students spent long hours using electronic devices [6]. E-learning, and thus prolonged sitting, is a topic that returned to higher education at the beginning of the 2022–2023 academic year. However, this change in learning methods is associated with musculoskeletal dysfunction including low back pain (LBP) [6,7].

LBP was one of the most common musculoskeletal disorders in the pre-pandemic period [8,9,10,11] and today it is known that the COVID-19 pandemic has increased the prevalence and intensity of LBP [6,7,10]. It should be emphasized the problem of LBP does not only apply to adults and the elderly. Teenagers and even children are increasingly struggling with serious back pain [9]. In the younger generation, this may be explained by sedentary behavior, prolonged sitting time, next to a reduced level of physical activity. The most common sedentary behavior, especially among adolescents, is the time spent sitting watching television, using the computer, playing video games, and using a smartphone [12].

Physical activity is significant for preventing LBP [13]. On the other hand, physical load during daily activities at work and during leisure time are defined as risk factors for LBP [14,15]. Exposure to physical load is not limited to one specific back-threatening activity but encompasses a compilation of activities, such as flexion, rotation, lifting, carrying, and pulling. Many popular exercises and positions with lumbar flexion may cause significant compression of the spine [16]. Prolonged sitting has been identified as a risk factor for LBP [17]. In most people, a relaxed sitting position is characterized by a loss of lumbar lordosis and a kyphotic position of the entire spine [18,19,20]. Such a position in the long-term results in several negative consequences within the musculoskeletal system. Prolonged sitting causes stretching of the supraspinous ligaments and a decrease in the activity of the multifidus muscle, which leads to limitations in its protective function for the spine. The load on the lumbar spine in the sitting position is significant. Disabling the proprioceptive function causes the load to be transferred to passive structures, such as ligaments and intervertebral discs, or passive muscle properties [21,22]. In addition, Williams et al. [23] showed that a kyphotic posture results in more back pain than a lordotic posture.

Re-education on sitting posture is a common aspect of LBP management. Physiotherapists recommend many different sitting positions to prevent and treat the negative effects of prolonged sitting. However, there is debate regarding what is an optimal sitting posture [18,24,25]. Studies showed that sitting posture with the backrest on the chair induced minimal changes in lumbar lordosis and significantly lesser pain compared to other types of the chair [26,27,28]. Each sitting position causes a static load on the spine. Shifting the load to other tissues can be achieved with a change in position. Repositioning is recommended to minimize the risk of tissue overload [21]. The systematic review of Waongenngarm et al. [29] showed that active breaks with postural changes were found to be effective in reducing discomfort and pain. Nonetheless, they suggested that there is insufficient real-life evidence (workplace contextualized) to provide more detailed information about the type of breaks and the break protocol [29]. On the other hand, others have investigated the effects of breaks on discomfort or pain in people at risk of LBP and excluded participants with LBP who reported pain in the last few months [29,30].

Investigations into the prevention and treatment of early symptoms of back pain are also worthwhile from an economic perspective because the direct costs of the treatment of individuals with chronic and recurrent LBP are high [31,32]. Therefore, it is very important to prepare evidence-based ergonomic recommendations—exercise protocols and interventions—for young people to reduce LBP and musculoskeletal discomfort during prolonged sitting, and consequently, to maintain health and quality of life. To our knowledge, these problems have not been fully investigated, especially among young people with LBP.

The study aims to evaluate the effectiveness of the active break program in reducing LBP and perceived musculoskeletal discomfort during prolonged sitting in young people with LBP.

## 2. Methods

This study will be a randomized controlled trial. The protocol was developed by the Recommendations for Randomized Trials (SPIRIT, Standard Protocol Items) [33]. Results will be reported as stated in the Consolidated Standards of Reporting Trials (CONSORT) statement [34]. This study is being conducted at the Faculty of Physical Education and Health, Jozef Pilsudski University of Physical Education in Warsaw, Biala Podlaska, Poland. The study was registered on clinicaltrials.org [NCT05810519] and complies with the latest version of the Helsinki Declaration [35]. This study was approved by the Ethics Committee of the AWF Warsaw [SKE 01-05/2023]. Written informed consent for participation will be obtained from the students.

### 2.1. Study Population

This study will include 1st- and 2nd-year students of a Bachelor’s course in Physical Education. Detailed information about participants will be given in the eligibility criteria.

#### 2.1.1. Inclusion Criteria

Participants will be recruited according to the inclusion criteria: female and male; age between 18 and 25 years; experiencing non-specific LBP (defined as pain and discomfort localized below the costal margin and above the inferior gluteal folds), without radiation to legs; not using medication for low back pain in the last 3 months; not having a surgical history due to spinal problems; not having radiculopathy or other injuries such as fractures, stenosis or tumors in the spine; lack of any treatment related to the low back within the last 6 months.

#### 2.1.2. Exclusion Criteria

Participants will be excluded from this study if they experience a level of low back pain of at least 7 on the 0–10 Visual Analogue Scale (VAS) scale (where 0 and 10 are “no pain” and “most severe pain I can imagine”, respectively); spinal pathology (e.g., tumor, infection, fracture, inflammatory disease); pregnancy; nerve root compromise; previous spinal surgery; major surgery scheduled during treatment or follow-up period; disc herniation; leg length discrepancy; pelvic asymmetry; and presence of any contraindication to exercise. In order to achieve homogeneity in terms of physical activity, professional athletes will be also considered ineligible.

### 2.2. Assessment

Before the assessment, written consent will be obtained from the participants. A blinded assessor, who is a physiotherapist with five years of clinical experience, will evaluate the eligibility criteria. The baseline assessment will be conducted before the random distribution of the participants into the groups. The assessment of the outcomes will be collected at baseline and immediately after the 12-week intervention.

### 2.3. Pre-Intervention Questionnaire

The survey contained questions regarding:

- gender,
- age,
- current physical activity (sports discipline, number of training days and hours per week),
- illnesses, injuries, surgical history due to spinal problems, treatment related to LBP, diagnosed defects in body posture,
- experience of LBP within the last three months. Individuals who respond positively (“yes”) to the question “Have you experienced low back pain for the last three months?” will then answer the question in the frequency of experiencing LBP (rarely, few times per week, often, or constantly) and the types of situations in which LBP occurred or increased.

### 2.4. Outcome Measures

The primary outcomes will be the frequency of LBP (questionnaire), pain intensity (VAS), disability index (ODI) and perceived musculoskeletal discomfort during prolonged sitting (Borg scale), assessed at baseline and after the 12-week intervention, and the Global Perceived Effect (7-point Likert scale), assessed only after the 12-week intervention. The secondary outcome will be the Post-Intervention Questionnaire (5-item self-completed questionnaire), assessed only after the 12-week intervention. Table 1 shows the distribution of the different measures across the time points during the present study.

All the tests will be performed during morning hours in the laboratory room at the Regional Centre for Research and Development in Biala Podlaska.

### 2.5. Primary Outcome Measures

#### 2.5.1. Pain Intensity

Average pain intensity will be assessed with the VAS. VAS is a validated, objective and unidimensional measure of pain [36]. Participants will be asked to rate their maximal pain intensity from the last 3 months on a 10 cm line. The centimeters will be marked by the participants and will be measured and classified according to the following key: 0—no pain, 1–3—mild pain, 4–6—moderate pain and 7–10—severe pain [37].

#### 2.5.2. Disability Index

The level of functional disability of participants resulting from LBP will be measured using the Revised Oswestry Low Back Pain Disability Index (ODI). The scale is one of the most reliable and effective outcome measures for evaluating patients with LBP [38]. The scale comprises 10 items questioning pain intensity related to activities of daily living. Each item provides six statements describing an increase in the level of severity of a particular activity, which is scored from 0–5 points. The total is calculated by multiplying the sum of the scores by 2, giving a range of 0 to 100 percent; a higher score reflects a higher disability [39].

#### 2.5.3. Perceived Musculoskeletal Discomfort during Prolonged Sitting

Perceived musculoskeletal discomfort during prolonged sitting will be measured using the Borg CR-10 scale during 1 h of sitting [40]. The body regions (i.e., the neck, shoulder, elbow, wrist, upper back, low back, buttocks, hip/thigh, knee, and ankle) will be defined according to a body chart from a modified Nordic questionnaire [41]. Participants will indicate which parts of their body experienced musculoskeletal discomfort and how much discomfort was felt (on a scale of 0–10; 0 denotes no discomfort and 10 denotes extreme discomfort).

#### 2.5.4. The Global Perceived Effect

The global perceived improvement will be assessed using The Global Perceived Effect Scale (GPE) [42,43]. It is a 7-point numerical scale (1–7). The participants will be asked: “Since the start of treatment, my current overall status is”: 1 = completely recovered, 2 = much improved, 3 = slightly improved, 4 = not changed, 5 = slightly worsened, 6 = much worsened and 7 = worse than ever. These ratings will be dichotomized into “improved” (GPE scores 1–2) and “not improved” (GPE scores 3 to 7) [44].

### 2.6. Secondary Outcome Measures

#### Post-Intervention Questionnaire

The postIQ will be focused on the opinions of the participants in relation to the active break program. The questions, among others, will be focused on perceived health, on the perceived improvement in knowledge of health-related topics, on the musculoskeletal symptoms during the program, on the usefulness of the content learned during the program, on the capability of incorporating the content learned during the program in daily living and on the exercises that were practiced at home. The items will be presented in the form of statements to which students will be asked to respond using a 1–5 Likert scale (“strongly agree”, “agree”, “not sure”, “disagree”, and “strongly disagree”).

### 2.7. Intervention Protocol

The intervention will be divided into the following two groups:

Group 1 (AB-group): participants will receive recommendations to take an active break with proposed lumbar and hip extension and hyperextension exercises for every 30 min of sitting.

Group 2 (C-group): participants will receive self-care recommendations and will be encouraged to remain active, performing their regular baseline activities.

### 2.8. Active Break Program

The experimental group will receive recommendations to take an active break every 30 min of sitting for 12 weeks. In the experimental intervention, the active break program will include lumbar and hip extension exercises and will be based on self-education with an explanation of the importance of regular training. The participants will receive a booklet with photographed and described exercises. The education will be carried out by an experienced physiotherapist.

The habit of doing exercises during an active break will be formed gradually (Table 2). In the first month, participants will be instructed to perform the proposed exercises in a standing position with lumbar and hip extension every 30 mins or whenever musculoskeletal discomfort occurs. In the second month, the complexity of the active break exercises will be increased by progressing to spine hyperextension exercise. In the third month, participants will be instructed to also perform spine hyperextension exercise in a sitting position. The number of repetitions depends on the needs of the participants.

### 2.9. Control Group

Participants assigned to the control group will receive an educational self-care book containing information on LBP, the anatomy of the spine and its relationship to the muscular chain, care during daily life activities and the importance of regular physical exercises. The control group will perform their regular baseline activities and will be advised to not alter their current behavior routine. Participants will be asked not to look for other treatments outside the trial during the intervention period.

Participants assigned to the control group will be offered the option of joining the exercise program with an active break after the final assessment has been completed.

### 2.10. Procedure

All 1st- and 2nd-year students of Physical Education will be informed of the research procedures, and those that are interested will be provided with a brief introduction to the selection process. One researcher from the university will conduct a pre-screening self-administered questionnaire (on paper) during classes with students. The pre-screening will address basic demographic data, the frequency of LBP, and the specific inclusion and exclusion criteria for this study. LBP will be assessed using the following question: “Have you experienced low back pain for the last 3 months?”. The answer options were: “no”, and “yes”. “Yes” was considered to denote the presence of LBP. A blinded assessor will evaluate the eligibility criteria. Participants who meet the inclusion criteria will be invited to the main study.

Before the baseline assessment, written consent will be obtained from the participants. At the baseline assessment session, participants will be requested to complete the VAS, Revised ODI, and Borg-10 scale. The baseline assessment will be conducted before the random distribution of the participants into the groups. After completion of the baseline assessment, participants will be randomly assigned into one of the two groups (AB-group or C-group), and simultaneous implementation of the two programs will commence. To improve adherence, participants from AB-group will send daily reports about hours in sitting positions and the number of active breaks via the Messenger^®^ application. Participants from C-group will be asked not to look for other treatments outside the trial during the interventions.

The assessment of the outcomes will be collected at baseline and immediately after the 12-week intervention.

### 2.11. Study Procedures

#### 2.11.1. Sample Size

The sample size was determined using the software G*Power 3.1, which used Factorial ANOVA with assumption of a medium-sized effect (d = 0.50) at a significance level of 0.05 and statistical power of 0.85. Therefore, the required sample size for this study will be 50 participants randomly assigned in two different groups. The sample will be increased by 20% to compensate for possible dropouts, leading to 32 individuals in each group (overall sample = 64 participants).

#### 2.11.2. Randomization

Randomization will be performed by a researcher not involved with recruiting and assessing the participants and will be generated via Microsoft Excel software 2019, before the beginning of the main study. Randomization will be stratified according to gender and the frequency of LBP. Treatment allocation will be concealed through sealed, opaque envelopes sequentially numbered. After the baseline assessment, one of the researchers responsible for the intervention will open the sealed envelopes to identify the group to which the participant was allocated: (1) with active breaks (AB-group); (2) with self-care recommendations (C-group).

#### 2.11.3. Blinding

The evaluation team will consist of four collaborators, one of whom will provide the interventions, one will evaluate the outcomes, one will randomize the participants and the last one will complete the statistical analysis. Double blinding will be used, with participant- and assessor-blinded. The participants will be blinded throughout the trial. All participants will be instructed that they will be allocated to one of the two protocols without knowing to which protocol. All the pre- and post-treatment assessments will be performed by a assessor blinded to the group allocation and treatment. The statistician performing the statistical analyses will also be blinded to the group allocation and treatment.

## 3. Study Participants Protocol Adherence

Participants are encouraged to adhere to the intervention. The intervention is determined based on the number of active breaks and adherence to recommendations. Commitment to the treatment group will be checked using an exercise log, text messaging and meetings. Every second day, the study coordinator will text participants and enquire about their protocol adherence or adverse events. Participants will also be encouraged to contact the study coordinator in case of any problems. Participants will receive positive feedback and reinforcement if they adhere to their intervention and return their exercise logs.

### 3.1. Statistical Analysis

The data will be analyzed using Statistica 14.0.0.15 program (TIBCO Software Inc., Palo Alto, CA, USA, 2020). The parameters will be described using basic descriptive statistical measurements, i.e., the percentage for qualitative variables, and the mean and standard deviation for quantitative variables. For variables that do not meet the criteria of normality of distribution, data will be presented as medians and quartile ranges. The normality of the distributions of the quantitative variables under study will be tested using the Shapiro–Wilk test. To verify the homogeneity of variances, Lavene’s test will be conducted. The chi-square test will be used for categorical variables. Descriptive statistics will be calculated separately for all groups (active break vs. control). The Student’s *t*-test (if data meet the criteria of normality of distribution) and the Mann–Whitney U test (if data do not meet the criteria of normality of distribution) will be used to compare parameter values between groups (active break vs. control). The Wilcoxon signed rank test will be used to examine the differences in parameters (VAS, ODI and discomfort) before and after the intervention. Intention-to-treat analysis will include all randomized patients. The significance level will be set at *p* < 0.05.

### 3.2. Missing Data

Lack of data may occur as a result of discontinuation of participation in the program or as a result of unexpected random events (e.g., illness). Since missing cases are expected to be less than 10% of the initial number of patients, the data will be analyzed as a full case study, excluding missing data from the analysis.

## 4. Discussion

This study has been designed to investigate the effect of active breaks on reduced LBP and perceived musculoskeletal discomfort during prolonged sitting in young people with LBP.

The SARS-CoV-2 infection has been shown to affect people’s health in multiple ways during active illness; furthermore, the long-term effects of this disease are also a growing concern [45]. The LBP appears to be a dominant and relatively persistent component of long-term COVID-19 [46]. Also sitting for a long time can cause discomfort in the low back or LBP [47]. In recent years, due to the flexibility of mobile devices, people are increasingly using electronic equipment without a desk [48]. When sitting without a back support or a desk, people often assume an incorrect sitting posture, which increases the risk of musculoskeletal disorders [49]. Although some epidemiological studies and systematic reviews have shown that sitting time and posture are not significantly associated with the development of LBP [50,51], other studies have shown that sitting with poor posture for long periods increases LBP and lumbar discomfort [52,53]. There is a lack of research studies that show ergonomic recommendations and effective exercises for reducing LBP during prolonged sitting in young people with LBP. To date, there have been an insufficient number of research studies that show ergonomic recommendations and effective exercises for reducing pain or discomfort during prolonged sitting in young people with LBP.

Prolonged sitting has been identified as a risk factor for LBP [17]. In sitting, the normal lumbar lordosis flattens and people sit in a more flexed lumbar position than they use during standing [20,54]. Most people sit for long hours in a slumped sitting posture [18,19]. The load on the lumbar spine in the sitting posture is not small and there is no adequate muscular support, as support is mainly switched off during the sitting position; as a result, passive structures like ligaments and intervertebral discs or passive muscle properties have to carry the load. Therefore, prolonged sitting causes changes in the passive structures of the spine and induces a low back compressive load significantly higher than that observed in standing [22].

Re-education on sitting posture is a common aspect of LBP management [28]. Increased discomfort from prolonged sitting has been attributed to increased muscle fatigue, decreased intervertebral disc nutrition, and reduced blood flow in muscles. Studies have shown that body-perceived discomfort scores increased over time during sitting and after 30 min of sitting are significantly greater than those at baseline [54,55].

Physiotherapists recommend many different sitting positions to prevent and treat the negative effects of prolonged sitting. However, there is debate regarding what is an optimal sitting posture. Periodic rest breaks have been identified as a way of reducing self-reported discomfort in the low back and standing breaks may be beneficial for the passive tissues of the spine [54,55]. A combination of sitting and standing is beneficial for reducing musculoskeletal discomfort [28]. Currently, breaks are recommended for mitigating the adverse of prolonged sitting, but more evidence is needed to verify the effectiveness of breaks. Waongenngarm et al. [29], in their systematic review, concluded that there is an insufficient evidence-base in this subject matter, especially concerning detailed break protocols [29]. However, a number of researchers have investigated the effect of breaks on healthy people and people if they were at risk of non-specific LBP [30]. It is important to spread more awareness regarding the correct work ergonomics while working in a sitting position at work, at university and home. People with LBP report significantly greater low back discomfort during prolonged sitting than their healthy counterparts [29,30]; however, further research is needed.

The strength of the study described here is the simple design and low-cost exercise training intervention. Students will perform exercises that will hardly be noticeable to the environment while sitting, what we called an active break. The active break program will be based on work- and home-based exercises. Self-management has an added value in the treatment of people with non-specific LBP. Work- or home-based exercises will save time and money, and are very convenient and hassle-free for participants. Facilitating people’s independence with LBP and providing tools that allow them to self-manage are generally considered important. Our main research outcome will be developed to match the needs of young people with LBP and the possibilities of the environment, to reduce LBP and musculoskeletal discomfort during prolonged sitting, and consequently, maintain health and quality of life. The intervention will address the social and health needs of a particular social group: young people, with the possibility of extending the recommendations to other groups. The research outcomes will build evidence-based practices for students, teachers, health professionals, personal trainers and family doctors.

We may face the major limitation of dropout of participants taking into account the length of the intervention (12 weeks). Therefore, to avoid a high dropout rate, we planned a system of rewards during the intervention and a gift for all students at the end of the final assessment. We cannot also exclude the occurrence of bias regarding monitoring the frequency and quality of sitting breaks each day. Other limitations that will arise during data collection and analysis will be identified and explained at the data presentation stage.

## 5. Conclusions

It is important to determine the effect of interventions that are feasible and effective in addressing LBP and perceived musculoskeletal discomfort in young people. To our knowledge, this is the first study examining the effect of active breaks with the proposed lumbar and hip extension exercises on reducing or decreasing LBP in students. We hope this study will help to develop ergonomic recommendations for young people with LBP.

## Figures and Tables

**Table 1:** SPIRIT table of enrolment, intervention, and assessment.

	Enrollment	Before Intervention	Randomization	Intervention
Timepoint	1st month	2nd month	2nd month	3rd, 4th, and 5th months (12 weeks)
Enrollment				
Eligibility pre-screening	+			
Informed consent	+			
Allocation			+	
Interventions				
Active break group and control group				+
Assessments				
Pain intensity		+		+
Disability index		+		+
Perceived musculoskeletal discomfort		+		+
The Global Perceived Effect				+
Post-Intervention Questionnaire				+

**Table 2:** Active break exercises.

	Exercises	Duration	
Phase 1—1st month	Spine extension in standing position: Extending arms above the head and reaching up. Straightening the torso until feeling the stretch in the lower spine. The range of arm extension depends on the mobility of the shoulder joints.	30 s	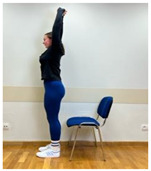
	2. Neutral standing position (lumbo-pelvic upright position)		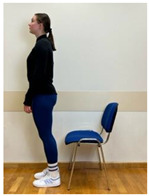
	3. Neutral sitting position (lumbo-pelvic upright position)		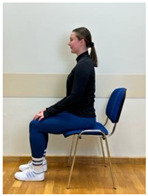
Phase 2—2nd month	Spine extension in standing position: Standing facing forward, feet on the floor. Extending arms above the head and reaching up. Straightening the torso until feeling the stretch in the lower spine. The range of arm extension depends on the mobility of the shoulder joints.	45 s	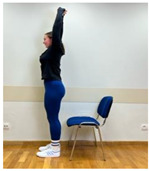
	2. Spine hyperextension in standing position: Putting arms behind the neck and keeping together elbows. Performing head retraction (hyperextension) and tilting your head back until feeling the hyperextension in the lower spine. This exercise is intended for participants who do not experience discomfort or increase in back pain caused by hyperextension in the lumbar lordosis. Participants sensitive to hyperextension perform this exercise with lumbar extension.		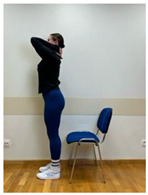
	3. Neutral standing position (lumbo-pelvic upright position)		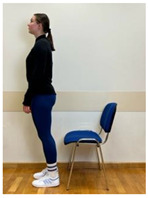
	4. Neutral sitting position (lumbo-pelvic upright position)		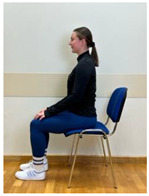
Phase 3—3rd month	Spine hyperextension in the sitting position: Putting arms behind the neck and keeping together elbows. Performing head retraction (hyperextension) and tilting your head back until feeling the hyperextension in the lower spine. The exercise is intended for participants who do not experience discomfort or increase in back pain caused by hyperextension in lumbar lordosis. Participants sensitive to hyperextension perform this exercise with lumbar extension.	60 s	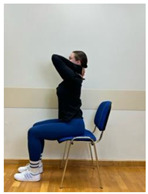
	2. Spine extension in standing position: Standing facing forward, feet on the floor. Extending arms above the head and reaching up. Straightening the torso until feeling the stretch in the lower spine. The range of arm extension depends on the mobility of the shoulder joints.		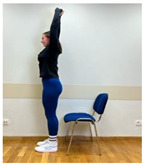
	3. Spine hyperextension in standing position: Putting arms behind the neck and keeping together elbows. Performing head retraction (hyperextension) and tilting your head back until feeling the hyperextension in the lower spine. The exercise is intended for participants who do not experience discomfort or increase in back pain caused by hyperextension in lumbar lordosis. Participants sensitive to hyperextension perform this exercise with lumbar extension.		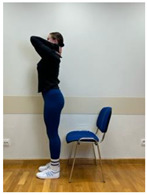
	4. Neutral standing position (lumbo-pelvic upright position)		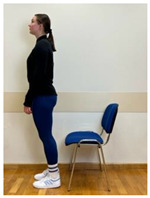
	5. Neutral sitting position (lumbo-pelvic upright position)		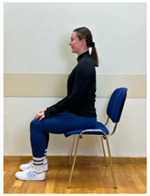

## Data Availability

Data sharing does not apply to this article as no datasets were generated or analyzed during the current study.
